# Supplementary figures and images for: Structural and functional analysis of vaccinia viral fusion complex component protein A28 through NMR and molecular dynamic simulations
Source: PLoS Pathog. 2023 Nov 10;19(11):e1011500. doi: 10.1371/journal.ppat.1011500 (PMC10664964; doi:10.1371/journal.ppat.1011500)

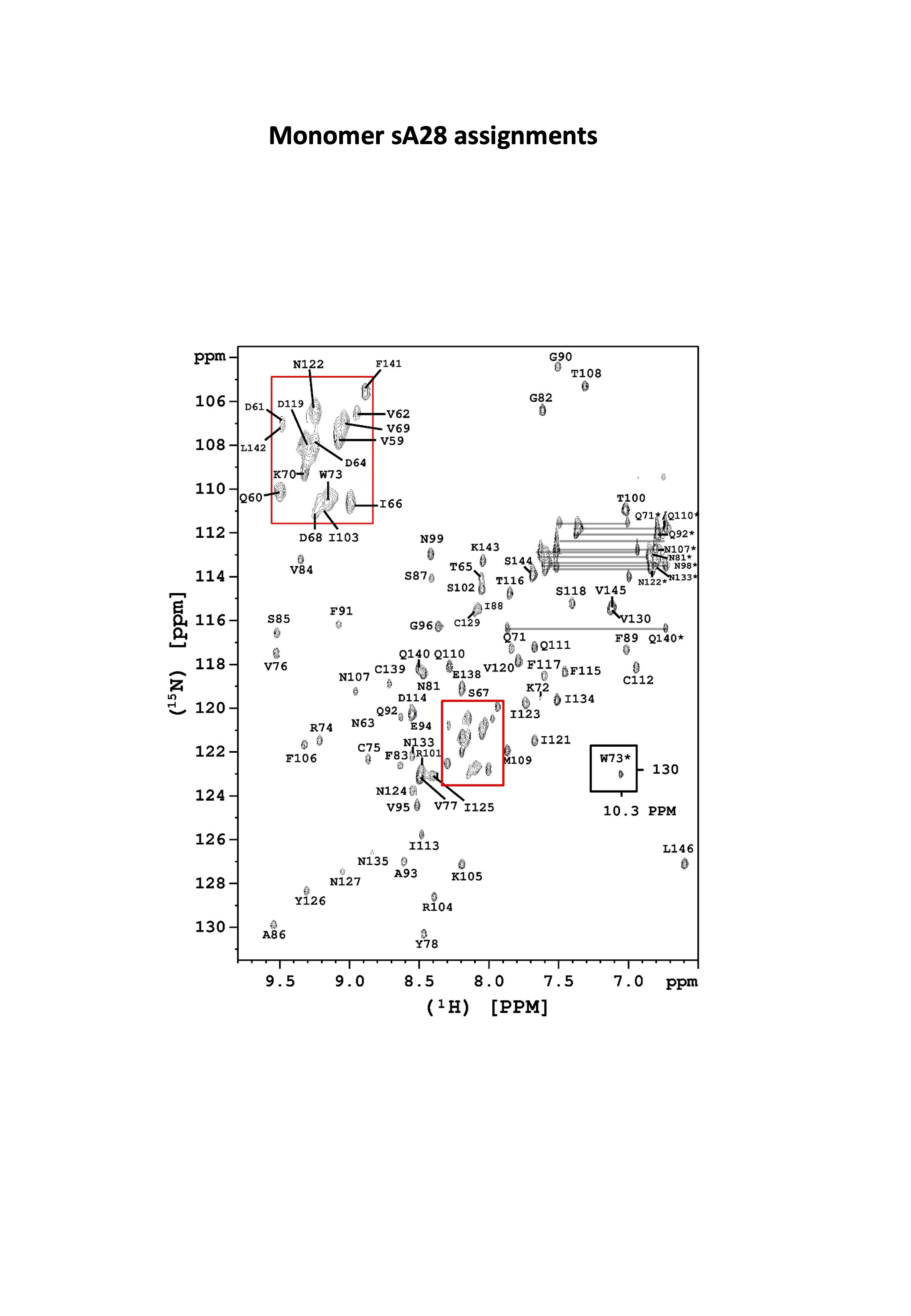

Supplement: S1 Fig — 2D 1H/15N HSQC spectrum of 15N-isotope-labeled sA28 (0.2 mM) at pH 6.5. The spectrum was collected at 25°C in an aqueous solution of 20 mM MES containing 50 mM NaCl. The assigned residues are indicated using single-letter codes. The insets show assignment details in the region with the maximum overlap of peaks (red box) and the region for the tryptophan side chain (black box). Assignments of the side-chain NH2 groups from the Asn and Gln residues are indicated with an asterisk, and horizontal gray lines connect the pairs of protons. (TIF) [file ppat.1011500.s001.tif]

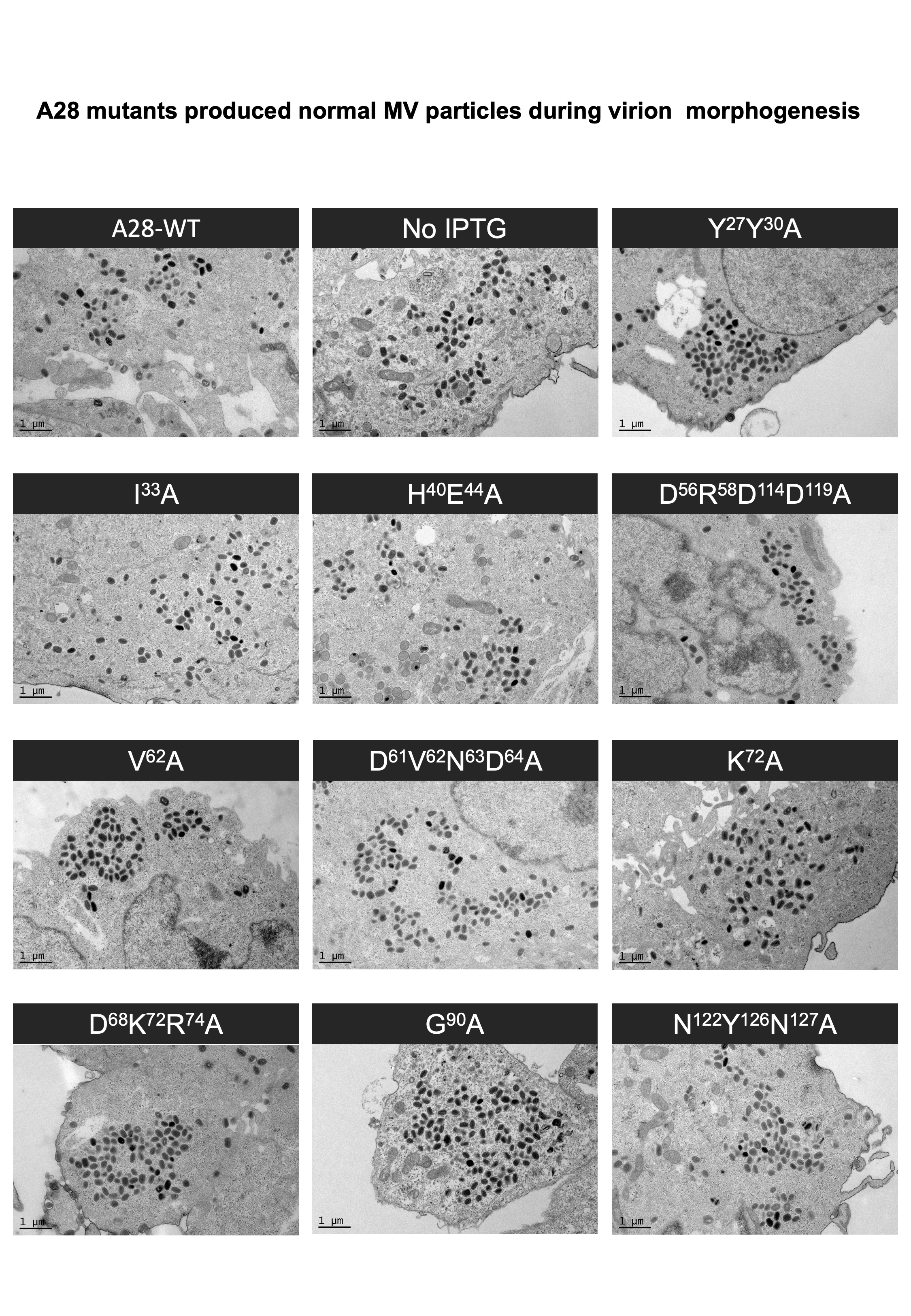

Supplement: S2 Fig — Confluent BSC40 cells in a 12-well plate were infected with viA28 and transfected with either control plasmid (No IPTG) or a plasmid expressing either wild-type or mutant A28 plasmids. At 24 hpi, cells were fixed, stained with uranylacetate, dehydrated and epon-embedded for sectioning and EM observation, as described previously [37]. The micrographs were photographed using a Tecnai G2 Spirit TWIN transmission electron microscope operating at 80 kV. All these infected cells produced abundant mature virus particles in cytoplasm with no blockage in virion morphogenesis. (TIF) [file ppat.1011500.s002.tif]

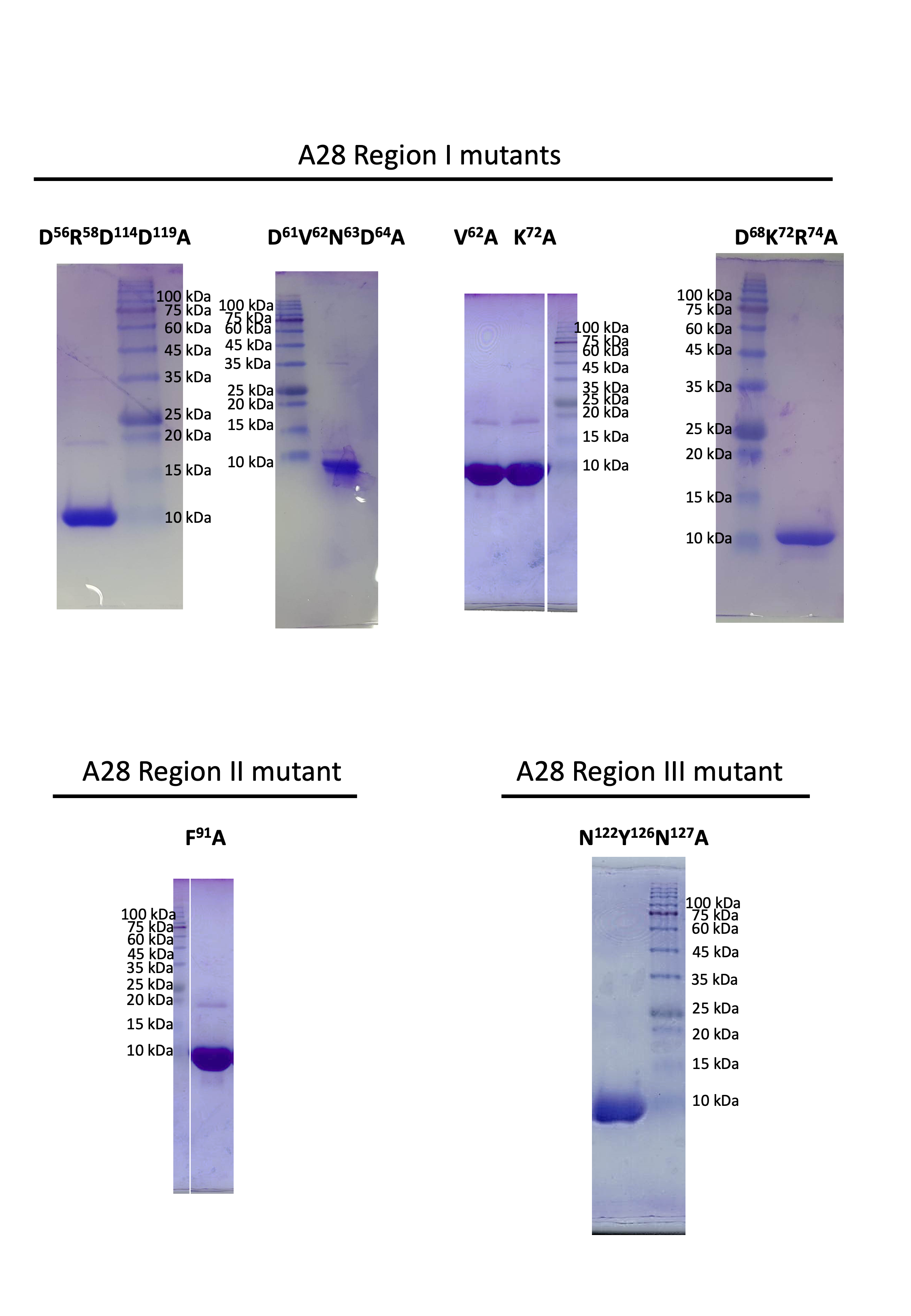

Supplement: S3 Fig — Coomassie blue staining of all the recombinant sA28 mutant proteins after purification (for ITC analyses in Fig 9). (TIF) [file ppat.1011500.s003.tif]

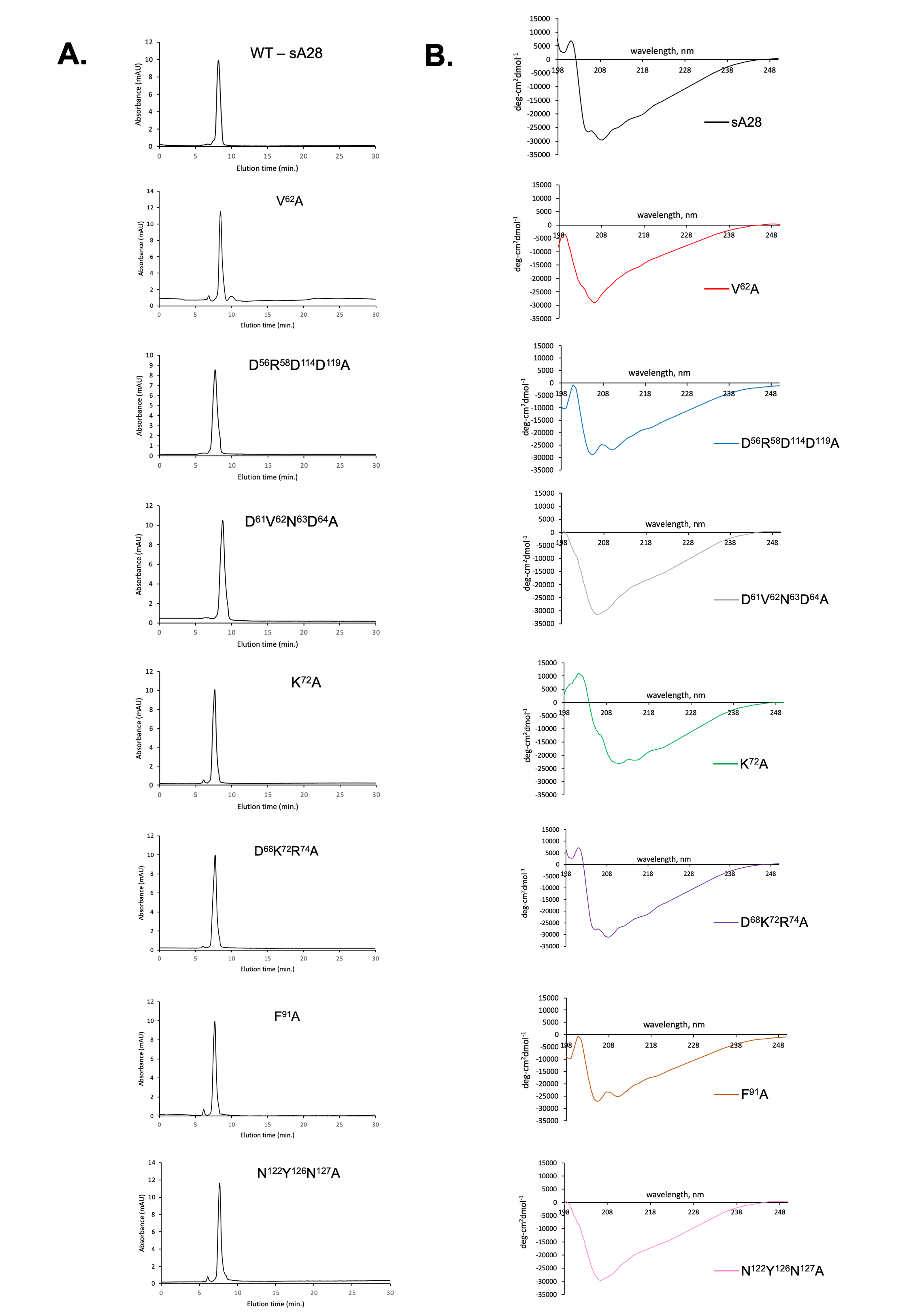

Supplement: S4 Fig — (A). Analyses of purified WT and mutant sA28 proteins using gel filtration chromatography. Gel-filtration chromatography profiles for all sA28 mutants were obtained with a TSKgel GMPWXL, 13 μm, all 7.8 mm ID x 30 cm x 4 column (Tosoh Bioscience, PA, USA) and a mobile phase composed of 20mM MES buffer (pH 6.5). All analysis were done at room temperature with a flow rate of 1.0 mL/min and UV detection at 280 nm. (B) Analyses of purified WT and mutant sA28 proteins using circular dichroism (CD) spectra analyses described in Materials and Methods. In brief, CD analysis was performed to monitor any aggregation or drastic changes in the secondary structure of sA28 mutant proteins when compared with the WT sA28. The CD spectrum of sA28 mutants (50 μM) in 20mM MES with 50mM NaCl at pH6.5 were recorded over the wavelength range from 250 to 190 nm, in 0.1 nm steps, as an average of 10 accumulations, using a 1 mm path-length quartz cuvette on a Jasco-815 spectrometer (Jasco Inc., Japan). All CD analyses were done at 25°C. (TIF) [file ppat.1011500.s004.tif]

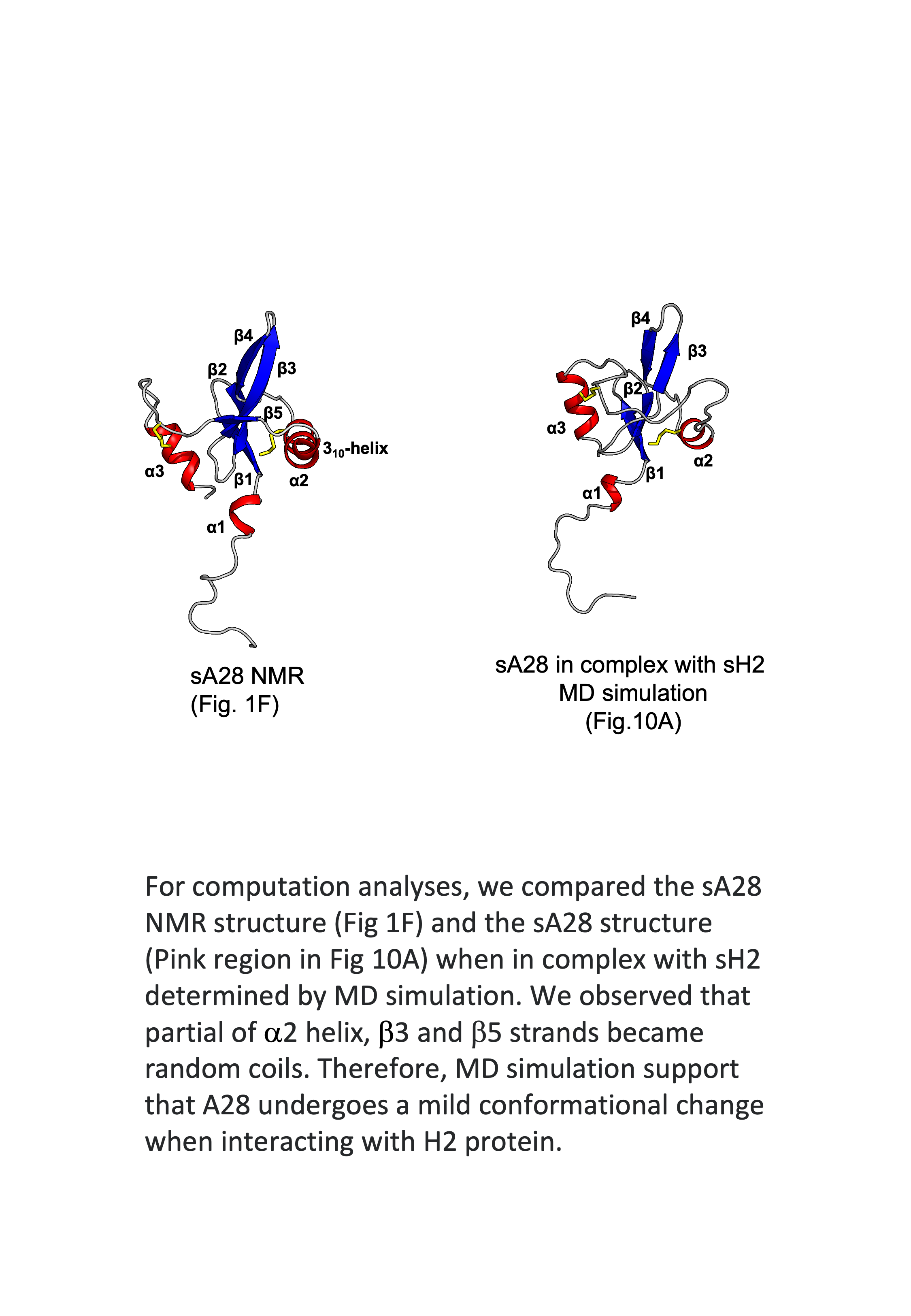

Supplement: S5 Fig — For computation analyses, we compared the sA28 NMR structure (Fig 1F) and the sA28 structure (Pink region in Fig 11A) when in a complex with sH2 determined by MD simulation. We observed that partial of α2 helix, β3 and β5 strands became random coils. Therefore, MD simulation suggested that A28 undergoes a mild conformational change when interacting with H2 protein. (TIF) [file ppat.1011500.s005.tif]

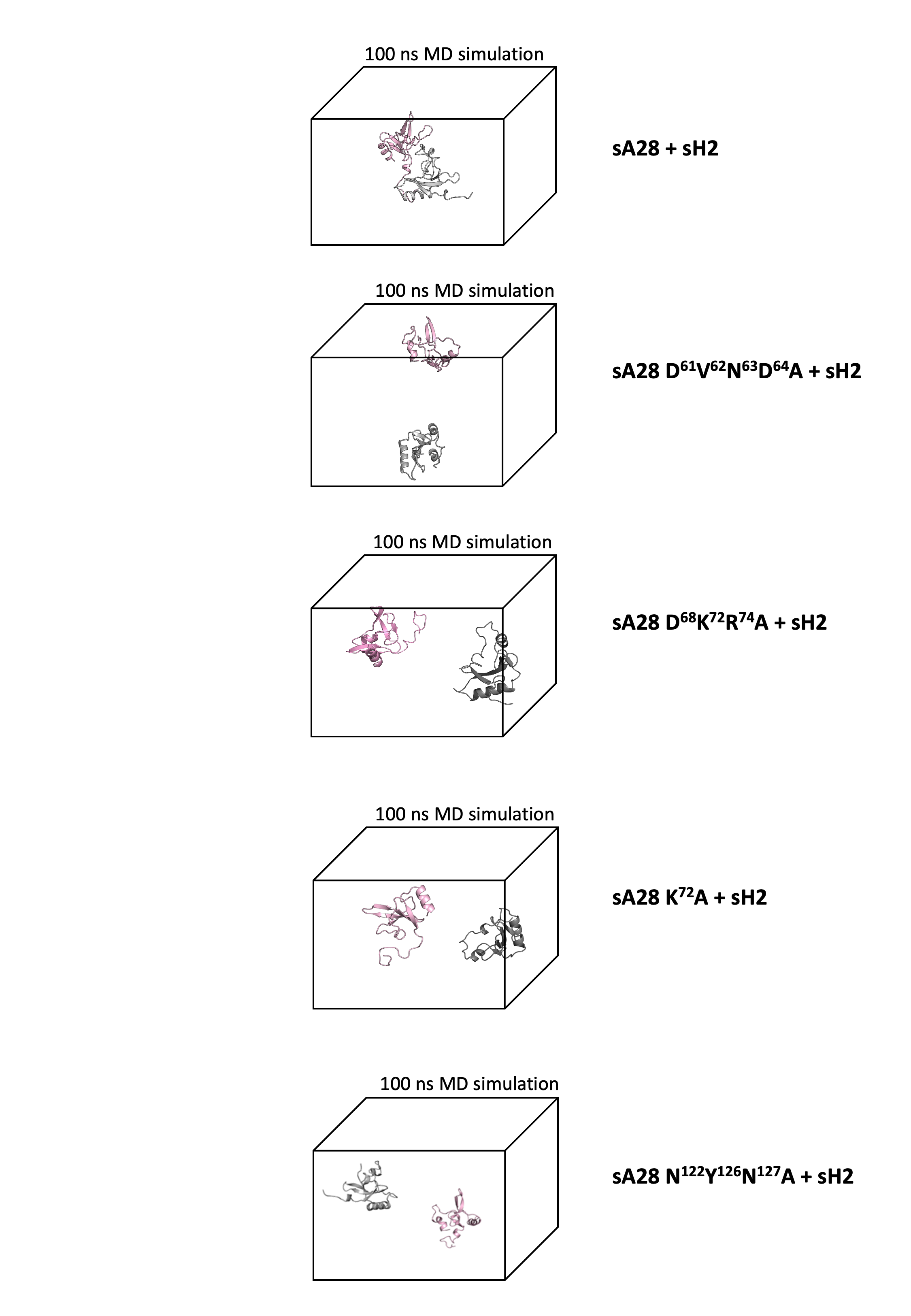

Supplement: S6 Fig — As described in the Materials and Methods, for the sA28-sH2 interaction, one molecule of sA28 and one molecule of sH2 were packed into a cubic box with dimensions ~300 Å using the software PACKMOL [63] to establish the initial point for the MD simulations. Following the same protocol, we conducted a MD simulation of wild-type sH2 in the presence of sA28 mutants, respectively. In contrast to the wild-type sA28 that forms a subcomplex with sH2, the MD simulation data revealed that the four sA28 mutants, i.e., three RI mutants: D61V62N63D64A, D68K72R74A and K72A, and one RIII mutant: N122Y126N127A, could not form a complex with sH2, indicating that these RI and RIII residues are indeed critical for the sA28/sH2 complex formation. (TIF) [file ppat.1011500.s006.tif]

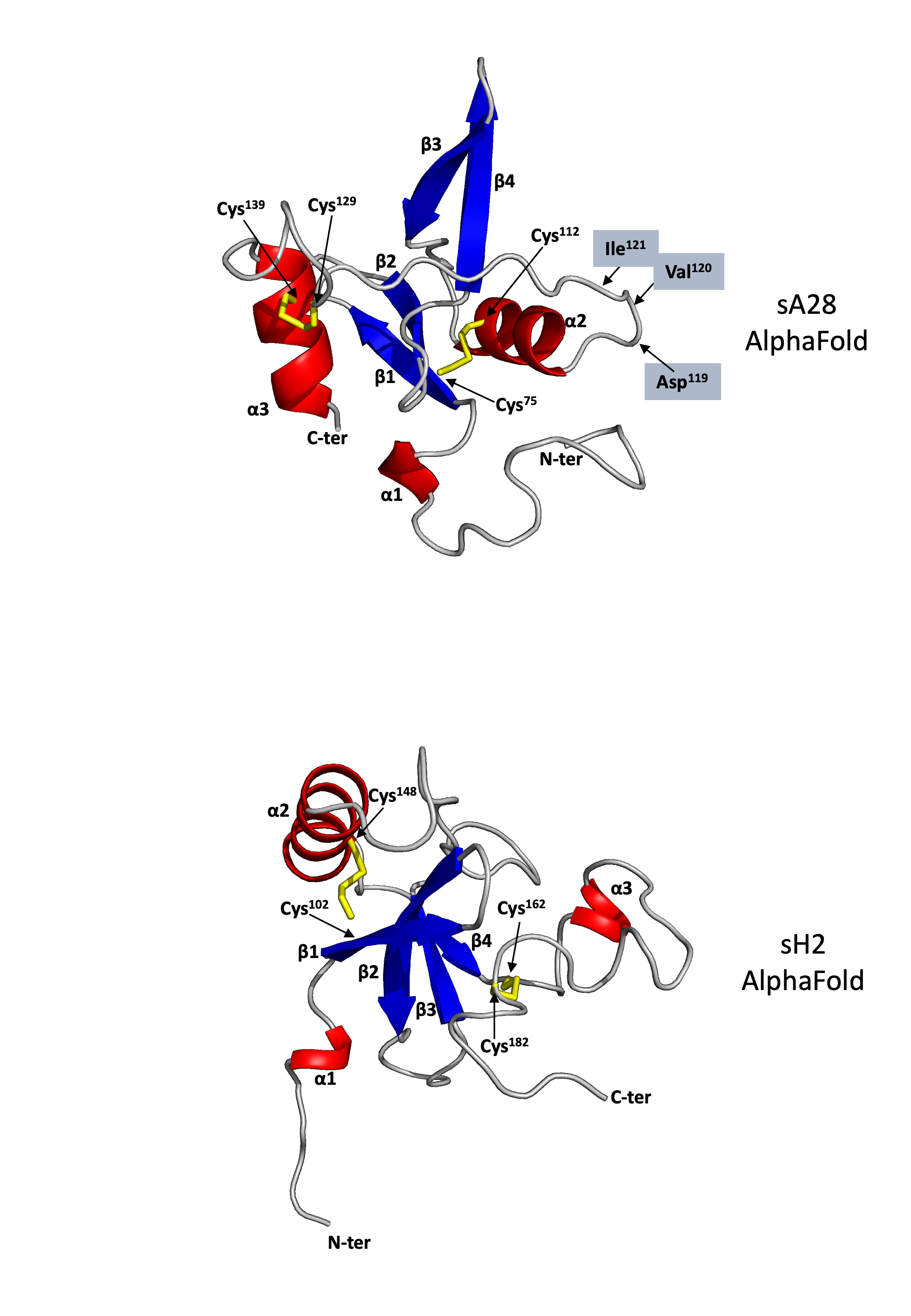

Supplement: S7 Fig — (TIF) [file ppat.1011500.s007.tif]

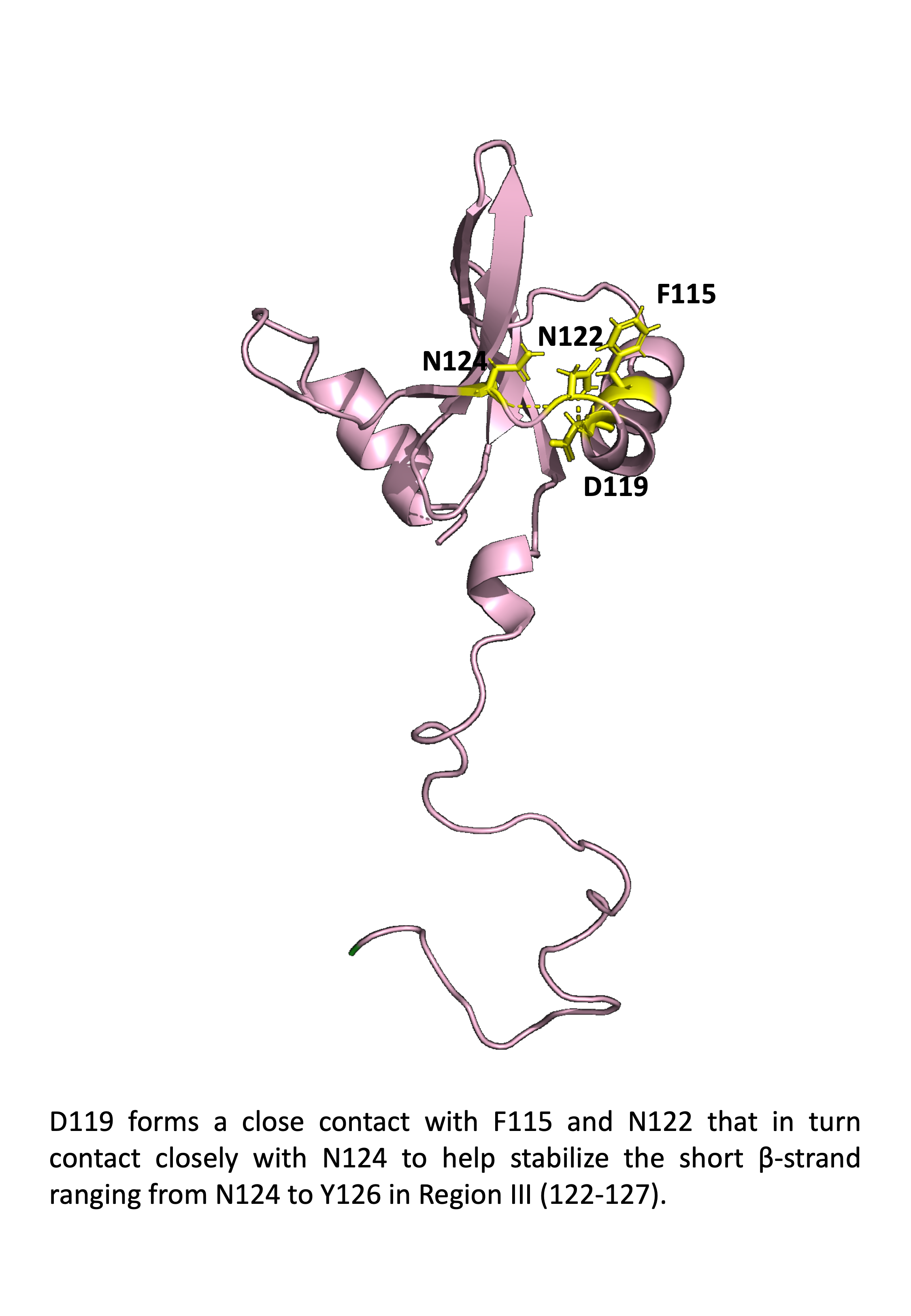

Supplement: S8 Fig — In the NMR structure of sA28 protein, D119 forms a close contact with F115 and N122 that in turn contact closely with N124 to help stabilize the short β-strand ranging from N124 to Y126 in Region III (122–127). (TIF) [file ppat.1011500.s008.tif]

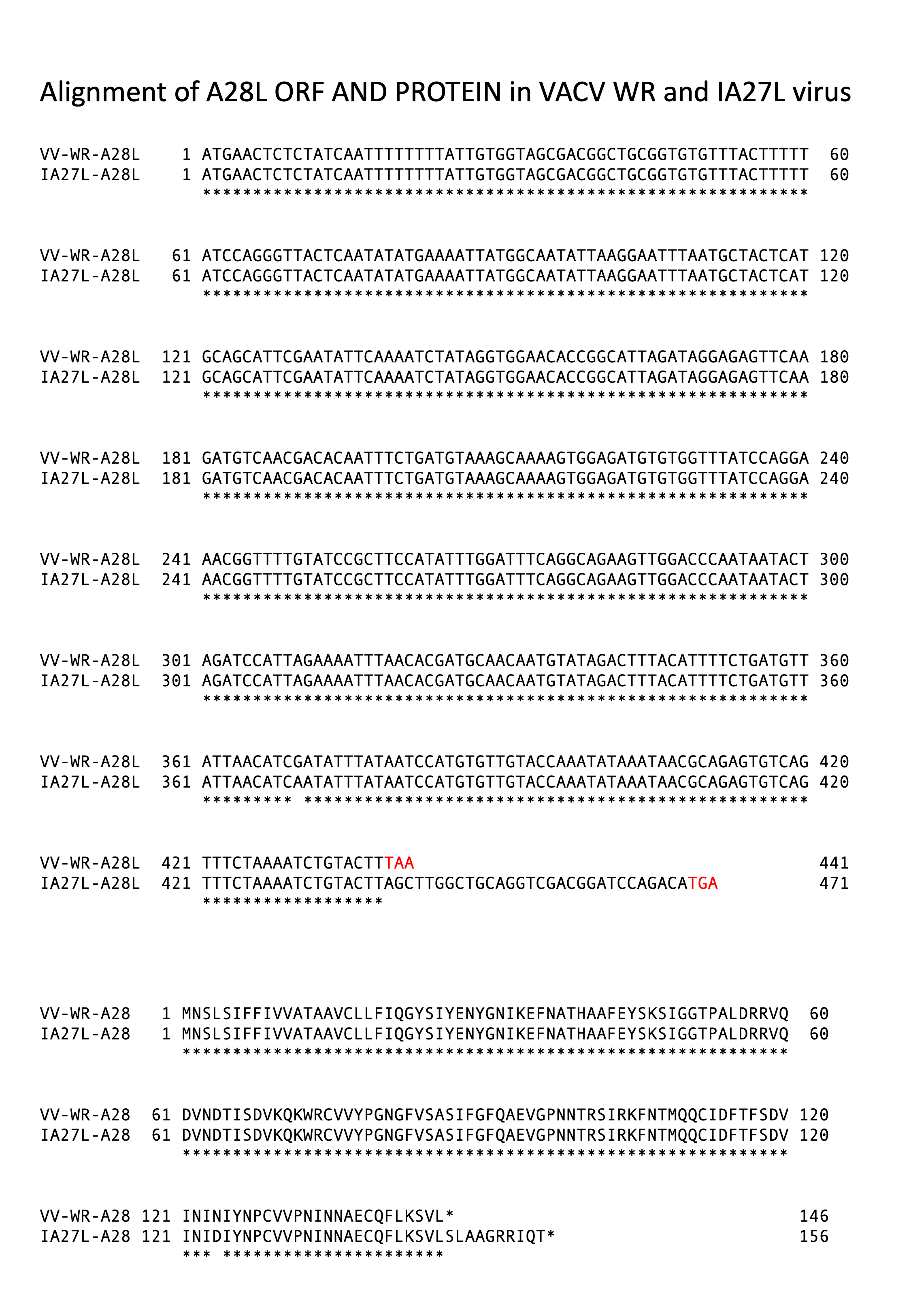

Supplement: S9 Fig — Alignment of A28L DNA and amino acid sequences of VACV WR and IA27L virus showed that the A28 protein encoded in IA27L virus contains 10 extra amino acids, SLAAGRRIQT, at the C-terminus that are absent in the WR virus. (TIF) [file ppat.1011500.s009.tif]

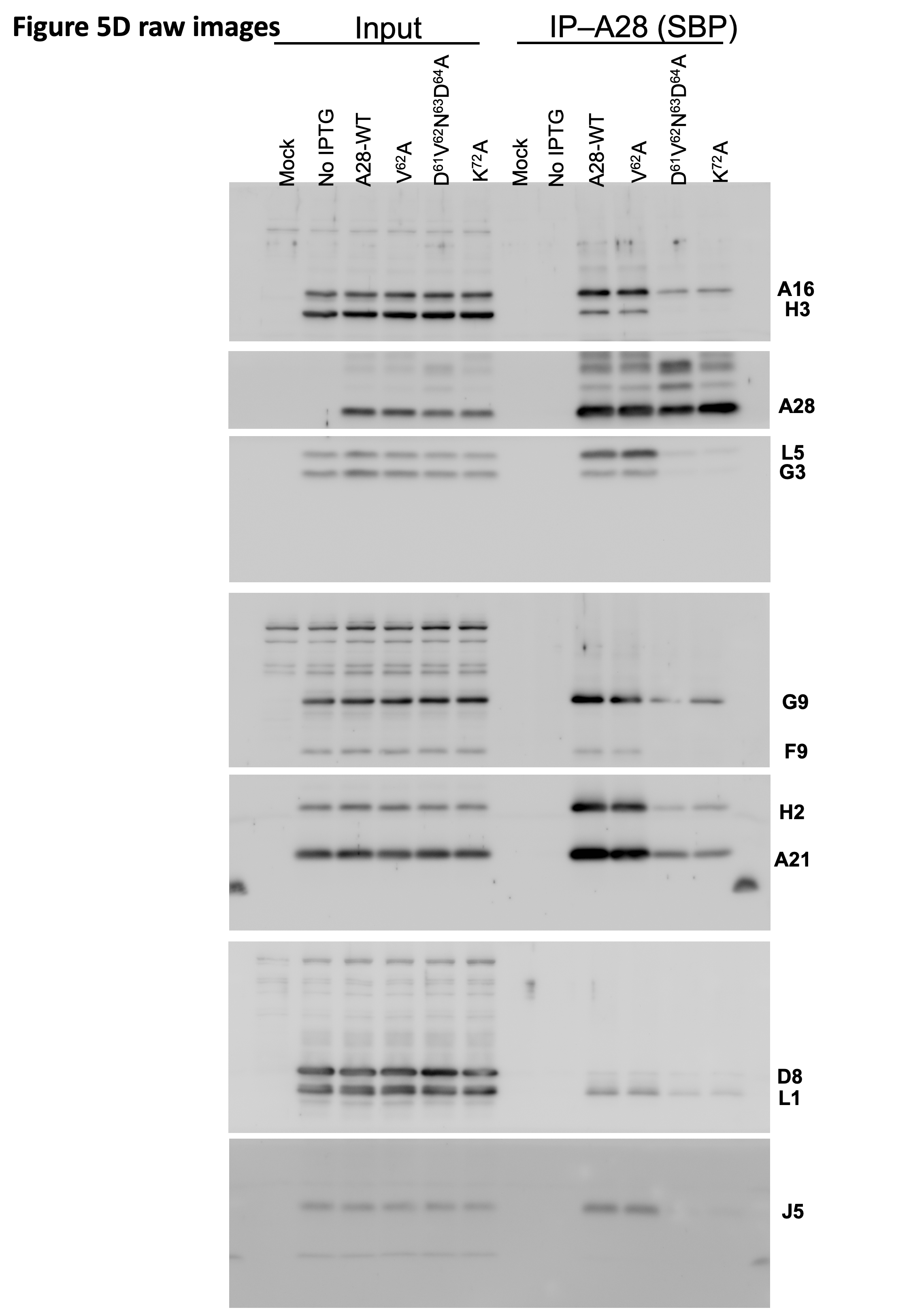

Supplement: S10 Fig — (TIF) [file ppat.1011500.s010.tif]

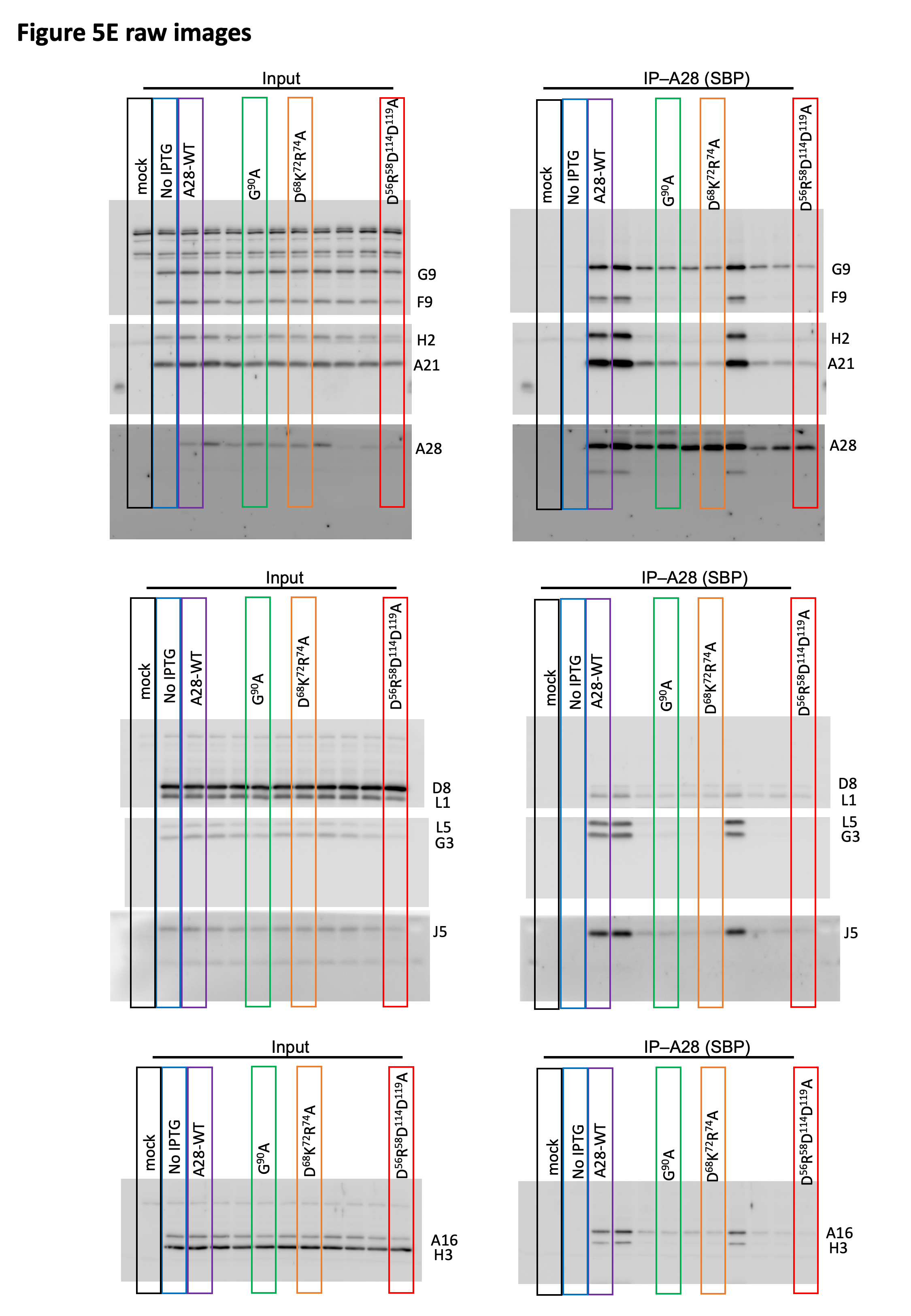

Supplement: S11 Fig — (TIF) [file ppat.1011500.s011.tif]

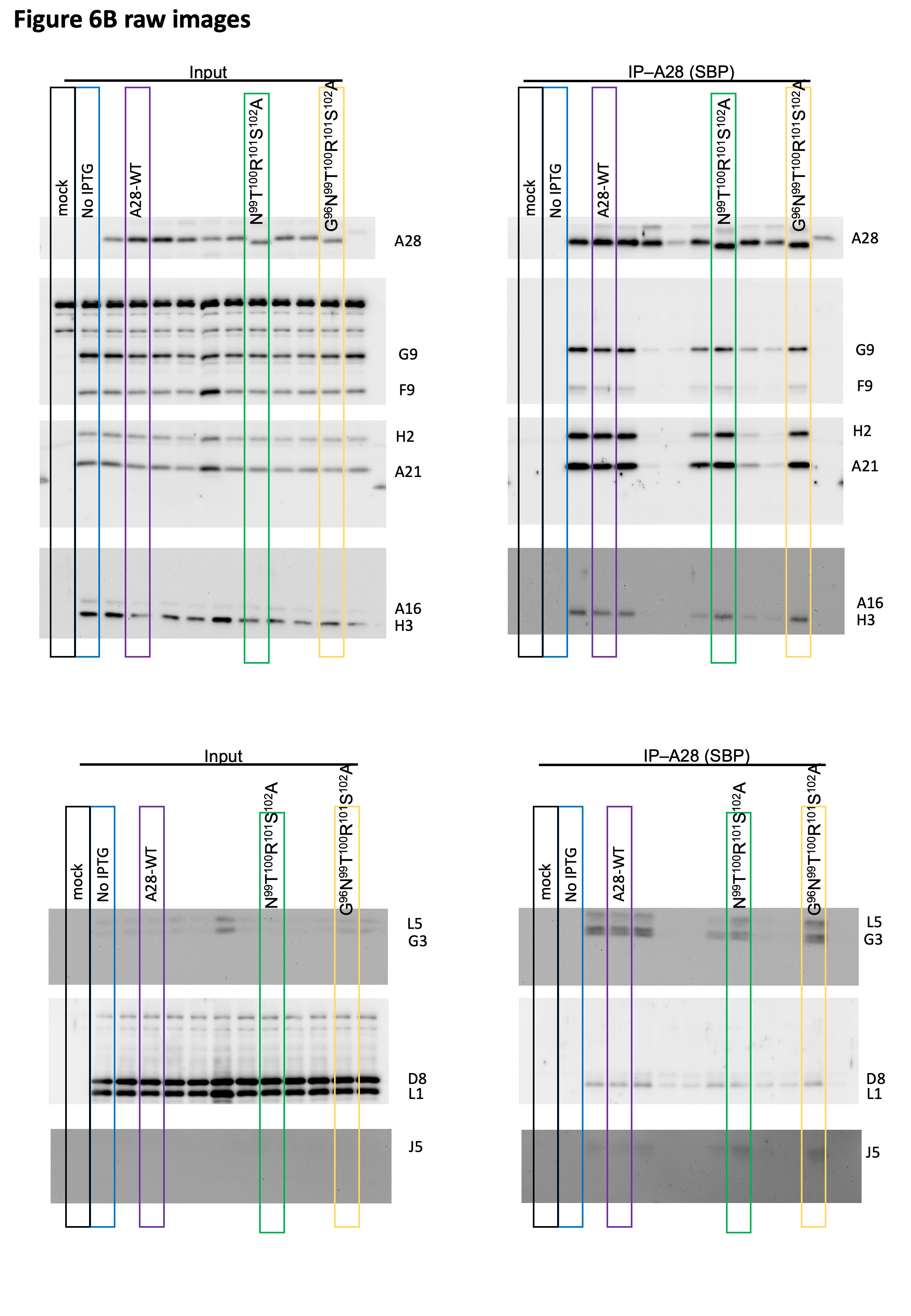

Supplement: S12 Fig — (TIF) [file ppat.1011500.s012.tif]

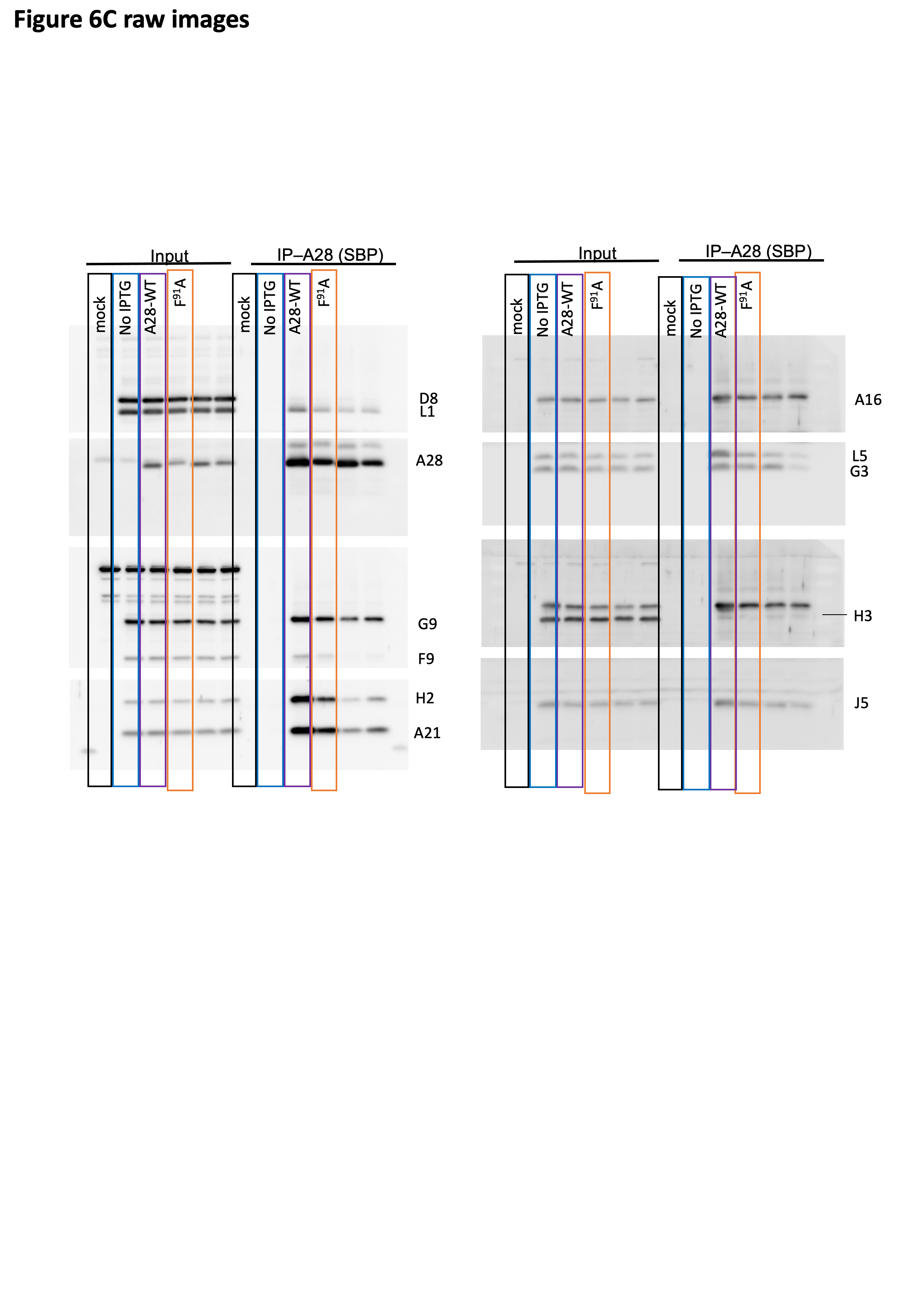

Supplement: S13 Fig — (TIF) [file ppat.1011500.s013.tif]

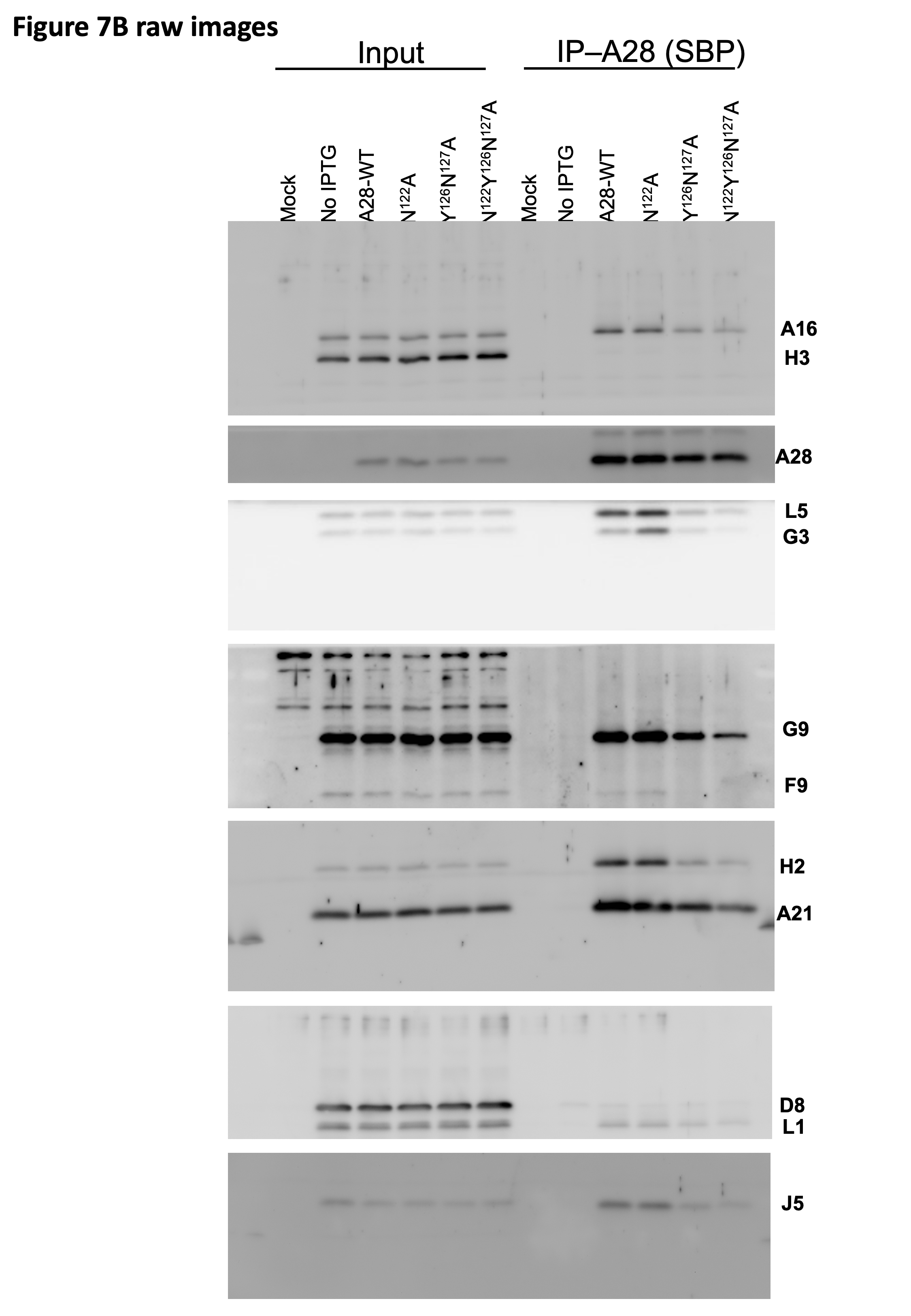

Supplement: S14 Fig — (TIF) [file ppat.1011500.s014.tif]

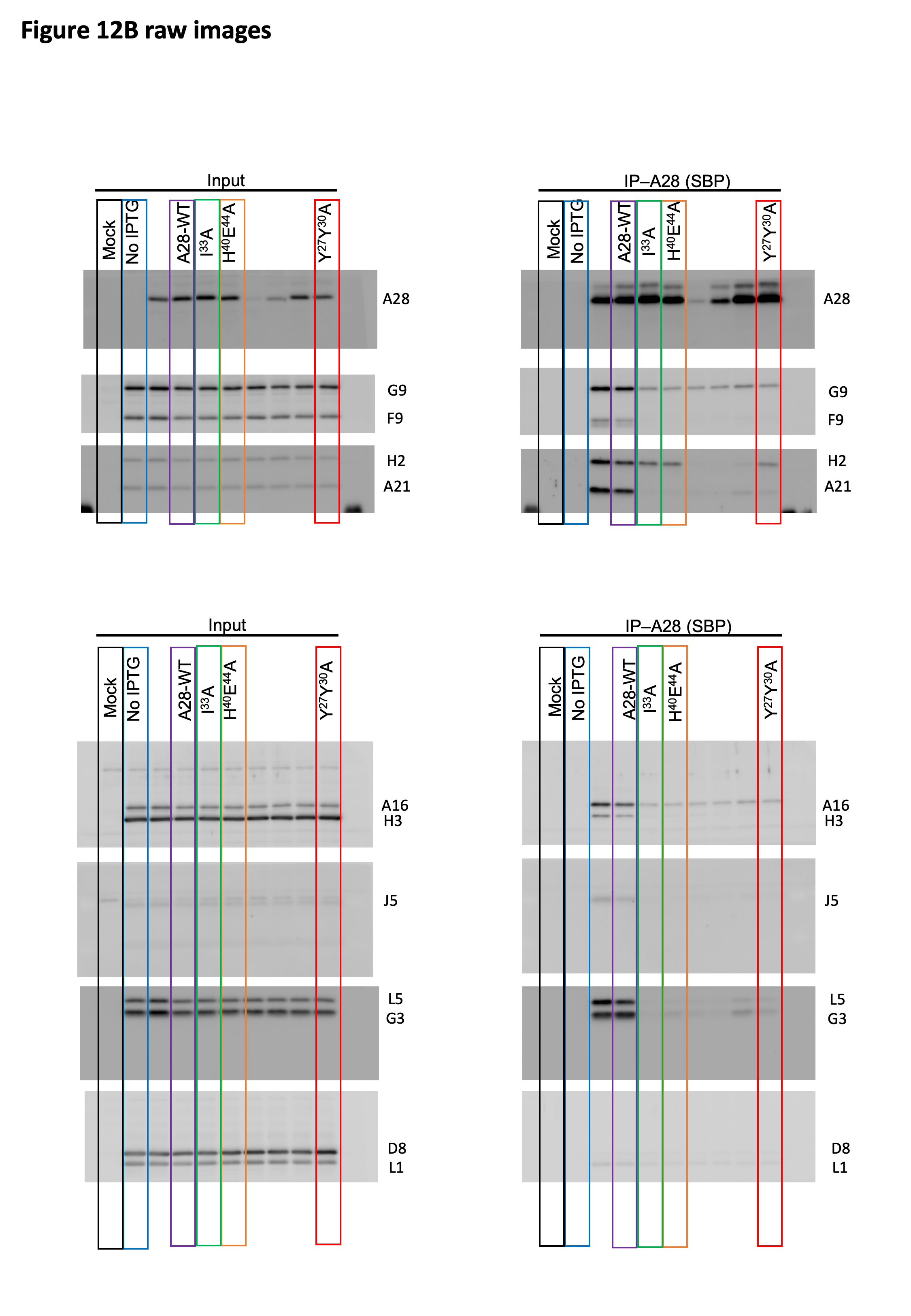

Supplement: S15 Fig — (TIF) [file ppat.1011500.s015.tif]
