# Supplementary material for: Structural and functional analysis of vaccinia viral fusion complex component protein A28 through NMR and molecular dynamic simulations
Source: PLoS Pathog. 2023 Nov 10;19(11):e1011500. doi: 10.1371/journal.ppat.1011500 (PMC10664964; doi:10.1371/journal.ppat.1011500)
Supplement: Attachment — Submitted filename: Item-by item response final 102723-4x.pdf [file ppat.1011500.s016.pdf]

Item-by-item responses to the reviewers' comments:

Part II – Major Issues: Key Experiments Required for Acceptance
Reviewer #1: None.
Reviewer #2: None noted.
Reviewer #3:
1. Can the authors provide some experimental or computational evidence on whether the A28 protein undergoes a conformational change after binding to H2. If there is indeed a conformational change in A28, the authors need to take it into account when performing molecular dynamic simulations.

Following the reviewer's suggestion, we conducted circular dichroism (CD) experiments (Fig 9) and computational analysis (S5 Fig) to check whether the sA28 protein undergoes a conformational change after the sH2 binding. Both data suggested that A28 may induce a mild conformational change in the secondary structure due to the sH2 binding. We have modified the text as shown below:
Line 278-288:
We wondered whether sA28 protein may induce a conformational change due to the sH2 binding. Since circular dichroism (CD) ellipticity at 208 and 222 nm are indicative of helical contents and that at 200 nm is sensitive to random coil in these proteins, we here measured the secondary structure of sA28 (Fig 9A) and sH2 proteins (Fig 9B), respectively, as well as sA28 and sH2 protein complex (1:1 molar ratio) (Fig 9C) by CD spectroscopy. A summation of the individual CD spectrum of sA28 and sH2 protein (Fig 9D) was overlaid with the CD spectrum of the sA28 and sH2 protein complex (Fig 9E). As resolved, the sA28 and sH2 protein complex formation gave rise to a weaker molar ellipticity in the range of 208 to 222 nm and a stronger molar ellipticity close to 200 nm (Fig 9E), indicating a slightly higher random coil content and less α-helical content. Thus, our CD measurements suggested that A28 may induce a mild conformational change in the secondary structure due to the sH2 binding.
Line 298-303:
When we compared the sA28 NMR structure (Fig 1F) and the sA28 structure in complex with sH2 as determined by MD simulation (Pink region in Fig 10A) we found that partial of α2 helix, β3 and β5 strands became random coils, suggesting that A28 undergoes a mild conformational change when interacting with H2 protein (S5 Fig). It is comforting that the MD simulation results are consistent with the CD data in Fig 9.
Line 532-538:
Circular dichroism spectra analyses of A28-H2 interaction
Circular dichroism analysis was performed to help identify any secondary structure perturbations occurring upon sA28 and sH2 binding. The CD spectrum of recombinant proteins sA28 (50 μM), sH2 (50 μM), and sA28-sH2 (1:1 mole ratio, 50 μM) complex samples in 20mM MES/50mM NaCl at pH6.5 were recorded over the wavelength range from 250 to 190 nm, in 0.1 nm steps, as an average of 10 accumulations, using a 1 mm path-length quartz cuvette on a Jasco-815 spectrometer (Jasco Inc., Japan). All CD analyses were done at 25°C.
Line 549-563:
For the A28-H2 interaction, one molecule of A28 and one molecule of H2 were packed into a cubic box with dimensions ~300 Å using the software PACKMOL [64] to establish the initial point for the MD simulations. This step ensured that no repulsive interactions would disrupt

or cause an error during the simulations. Using a V-rescale thermostat, the overall temperature of water and protein molecules was kept constant by coupling each group of molecules independently at 300 K. A Parrinello-Rahman barostat [65] was used to couple the pressure to 1 atm in every dimension separately. The time constants for the temperature and pressure couplings were 0.1 and 2 ps, respectively. A time step of 2 fs was achieved using the leapfrog algorithm to integrate equations of motion for the system. Periodic boundary conditions were also set for the whole system. We set a 1 nm cut-off for the Lennard–Jones and Ewald summation of Coulombic interactions. The Fourier space element of Ewald splitting was calculated using the Particle-Mesh-Ewald method [66] by applying cubic spline interpolation and a 0.16 nm grid length on the side. A TIP3P water model [67] was used, and the protein parameters were obtained from the AMBERff99SB-ILDN force field [68]. MD simulations were done for a total of 100 ns scan length.

Line 875-881:

S5 Fig. Comparison of sA28 NMR structure with sA28 in complex with sH2 determined by MD simulation.

For computation analyses, we compared the sA28 NMR structure (Fig 1F) and the sA28 structure (Pink region in Fig 10A) when in a complex with sH2 determined by MD simulation. We observed that partial of α2 helix, β3 and β5 strands became random coils. Therefore, MD simulation suggested that A28 undergoes a mild conformational change when interacting with H2 protein.

2. We know that manually incorporating mutation(s) on a protein may cause its instability or aggregation. In the in-vitro ITC assays (Figure 8A, 8B and 8C), the authors prepared several mutants of the sA28 protein but lacked the solution-behavior characterization of them. Therefore, more data (such as gel-filtration-chromatography profiles and circular-dichroism-spectra figures, etc.) need to be provided to show that these protein samples are homogeneous.

Following the reviewer's suggestion, we performed gel filtrations (S4A Fig) and CD analyses (S4B Fig) for the wild-type sA28 protein and seven mutant sA28 proteins described in Fig 8, $V^{62}A$, $D^{56}R^{58}D^{114}D^{119}A$, $D^{61}V^{62}N^{63}D^{64}A$, $K^{72}A$, $D^{68}K^{72}R^{74}A$, $F^{91}A$, and $N^{122}Y^{126}N^{127}A$. These results demonstrated that the wild-type and all seven mutant A28 proteins are soluble and homogenous. We have modified the text as shown below:

Line 262-264:

Using gel filtration chromatography (S4A Fig) and circular-dichroism-spectra analyses (S4B Fig) these proteins exhibited homogeneous profiles.

Line 862-874:

S4 Fig. sA28 mutant proteins are homogenous.

(**A**). Analyses of purified WT and mutant sA28 proteins using gel filtration chromatography. Gel-filtration chromatography profiles for all sA28 mutants were obtained with a TSKgel GMPWXL, 13 µm, all 7.8 mm ID x 30 cm x 4 column (Tosoh Bioscience, PA, USA) and a mobile phase composed of 20mM MES buffer (pH 6.5). All analysis were done at room temperature with a flow rate of 1.0 mL/min and UV detection at 280 nm.

(**B**) Analyses of purified WT and mutant sA28 proteins using circular dichroism (CD) spectra analyses described in Materials and Methods. In brief, CD analysis was performed to monitor any aggregation or drastic changes in the secondary structure of sA28 mutant proteins when compared with the WT sA28. The CD spectrum of sA28 mutants (50 µM) in

20mM MES with 50mM NaCl at pH6.5 were recorded over the wavelength range from 250 to 190 nm, in 0.1 nm steps, as an average of 10 accumulations, using a 1 mm path-length quartz cuvette on a Jasco-815 spectrometer (Jasco Inc., Japan). All CD analyses were done at 25°C.

Part III – Minor Issues: Editorial and Data Presentation Modifications
Please use this section for editorial suggestions as well as relatively minor modifications of existing data that would enhance clarity.

Reviewer #1: 1. The rationale for targeting the specific A28 residues for mutagenesis is not explained clearly. There are many residues in region 1, 2,3. Why did you choose the specific subset of residues for mutagenesis? For example, why do you generate the D56R58D114D119A mutation, which also targets the a2 helix?

In response to the reviewer's comment, we have modified the text as shown below:

Line181-190:

When we performed mutagenesis designs, we postulated that surface-exposed residues would constitute domains accessible for protein-protein interaction. Therefore, we generated seven A28 mutant constructs containing changes at single or multiple surface amino acids in RI (aa59-72), three mutants in RII (aa91-105) and three mutants in RIII (aa122-127) (Fig 4A). In the full-length A28 structure modeled by AlphaFold, D114D119 appear on the protein surface close to D56R58 in RI so we generated not only the D56R58A RI mutant but also the D56R58D114D119 RI mutant. Furthermore, we picked four highly conserved residues, W73, R74, F89 and G90, that are outside of the RI, RII and RIII regions (Fig 4B) as targets to generate four conserved mutants (Fig 4A). In total, we generated 17 mutant plasmids.

2. Line 187: "However, after careful modifications of plasmid transfection conditions." The specific modification is not described anywhere in the text.

In response to the reviewer's comment, we have modified the text as shown below:

Line 592-597:

For mutants that showed diminished A28 expression, $D^{56}R^{58}D^{114}D^{119}A$, $D^{68}K^{72}R^{74}A$ and $G^{90}A$, the transfection condition was modified as the following: we reduced the transfected wild-type A28 expression plasmid DNA from 0.05 µg to 0.01 µg and increased the A28 mutant expression plasmid DNA from 0.05 µg to 2 µg per well to achieve comparable A28 protein expression level between the wild type and the mutant A28 protein of interest.

3. Around line 255. The H2 structure used for molecular dynamics analysis is an alphafold predicted structure. It should be stated as such in the result section

In response to the reviewer's comment, we have modified the text as shown below:

Line 298:

...of A28 and the Alphafold-predicted H2 (S7 Fig), i.e., sA28 and sH2. ....

.

4. Line 550-556. The method for fusion from without experiment appears to be incorrect. Why were the cells incubated with inf/tf lysates overnight?

Sorry about the confusion. We corrected the text as shown below:

Line 632-636:

The next day, these cells were pre-treated with 40 μg/ml cordycepin for 1 hour at 37°C and subsequently infected with the inf/tnf cell lysates harvested from transient complementation assays. After 1 hour of incubation, the viral inoculum was removed and the cells were washed with warm PBS and then incubated in PBS (pH7.0) or PBS (pH5.0) at 37°C for 3 minutes.

5. Line 640. There is an extra B in the text.

Removed.

6. Figure 1D. There is a beta5 strand in the figure, but the text only describes 4 beta strands.

We thank the reviewer for pointing out the mistake. The text has been modified to include the β5 strand as shown below:

Line 114-115:

and five anti-parallel β-strands-β1 (74-78), β2 (82-87), β3 (90-96), β4 (100-103) and β5(124-126)—with the remaining residues forming connecting loops (Fig 1C).

Line 128:

β-strands: 74−78, 82−87, 90−96, 100−103 and 124-126.

Line 660-661:

five β-strands: β1 (aa 74–78), β2 (aa 82–87), β3 (aa 90–96), β4 (aa 100–103) and β5 (aa 124–126);

7. Figure 2D. Region 2 does not have signal decay and was later shown not important for H2 binding. Perhaps the data in Figure 2D is a better predictor for H2 binding region?

Indeed. We added some discussion in the text as shown below:

Line 391-395:

One possible explanation for this discrepancy is that the chemical shift for RII in the 2D HSQC data (Fig 2C) may be an indirect consequence of A28-H2 subcomplex formation rather than contributing to A28-H2 interaction. Instead, diminished signal intensities derived from the 2D HSQC spectra (Fig 2D) predicted better for A28-H2 binding regions.

8. Figure 6. For N99T100R101S102A mutation, cell fusion and co-IP experiments are not performed.

In response to the reviewer's request, we performed the cell fusion and co-IP experiments on this A28 mutant. The data are included in Fig 5B (cell fusion image), Figure 5C (quantification data of cell fusion), and Fig 6B (Co-IP).

9. Figure 7. For N122A and Y126N127A mutations, cell fusion and co-IP experiments are not performed.

In response to the reviewer's request, we performed the cell fusion and co-IP experiments of these two A28 mutants. The data are included in Fig 5B (cell fusion image), Figure 5C (quantification data of cell fusion), and Fig 7B (Co-IP).

Reviewer #2: 1. Is A28 thought to be mainly a structural protein and involved in complex formation? There do not appear to be A28 mutations that affect fusion without affecting complex formation (thus far). Presumably, one or more of the EFC proteins will contain domains involved in membrane fusion but perhaps not directly in complex formation. Mutations with the aforementioned attributes would lead to a better idea about membrane fusion outside of pure complex formation. This idea could be discussed.

We agree with the reviewer's comments and have modified the discussion as shown below:

Line 444-455:

Previous studies suggested that A28 protein is essential for viral EFC formation [10, 55] and that mutant vaccinia MV particles devoid of A28 protein can initiate hemifusion between host and viral membranes [25]. Our A28 mutagenesis results appeared consistent with that idea since all the A28 mutants that have lost MV infectivity are also defective in EFC assembly. Taken together, these data imply that a stable viral EFC structure, but not the membrane fusion step, requires A28 protein. Since vaccinia EFC contains 11 component proteins presumably one or more of the other EFC proteins may directly participate in the membrane fusion step. Clearly, identifying which component(s) of vaccinia EFC serves as the canonical fusion protein(s) to initiate membrane fusion will be the most exciting research direction in the coming years. One may anticipate that mutations of a candidate viral fusion-genic domain in EFC will lead to a fusion-defective phenotype without interrupting viral EFC formation.

2. Were H2/A28 MD simulations repeated with any A28 mutants where complex formation was disrupted? What would be the expected result?

In response to the reviewer's request, we performed the MD simulations of sH2 binding with wild-type and A28 mutants, including three RI mutants $D^{61}V^{62}N^{63}D^{64}A$, $D^{68}K^{72}R^{74}A$, $K^{72}A$, and one RIII mutant $N^{122}Y^{126}N^{127}A$. The wild-type sA28 and sH2 could form a subcomplex; however, sH2 could not form a complex with these A28 mutants (S6 Fig), indicating that these A28 RI and RIII residues are critical for the sA28/sH2 complex formation. We have included these results and modified the text as shown below:

Line 311-315:

We also performed MD simulations with the loss-of-function RI and RIII mutants of sA28, such as $D^{61}V^{62}N^{63}D^{64}A$, $D^{68}K^{72}R^{74}A$, $K^{72}A$ and $N^{122}Y^{126}N^{127}A$, to determine whether they can interact with sH2. As expected, when wild type sA28 protein formed a complex with sH2 in a total of 100ns scan length, all these sA28 mutant proteins failed to interact with sH2 protein (S6 Fig).

Line: 882-891:

S6 Fig. The MD simulations of wild-type sH2 in the presence of sA28 mutants.

As described in the materials and methods, for the sA28-sH2 interaction, one molecule of sA28 and one molecule of sH2 were packed into a cubic box with dimensions ~300 Å using the software PACKMOL [64] to establish the initial point for the MD simulations. Following the same protocol, we conducted a MD simulation of wild-type sH2 in the presence of sA28 mutants, respectively. In contrast to the wild-type sA28 that forms a subcomplex with sH2, the MD simulation data revealed that the four sA28 mutants, i.e., three RI mutants:

$D^{61}V^{62}N^{63}D^{64}A$, $D^{68}K^{72}R^{74}A$ and $K^{72}A$, and one RIII mutant: $N^{122}Y^{126}N^{127}A$, could not form a complex with sH2, indicating that these RI and RIII residues are indeed critical for the sA28/sH2 complex formation.

3. Alpha fold structural predictions are mentioned in the discussion. It would be of interest to show those for A28 and H2, especially to provide support for certain domains that seem to have structural functions. Could be placed in the supplement if high quality predictions can be obtained.

In response to the reviewer's request, we have included the AlphaFold structural predictions of sA28 and sH2 in the S7 Fig and modified the text as shown below:

Line 298:

...of A28 and the alpha fold-predicted H2 (S7 Fig), i.e., sA28 and sH2. ....

Line 374-379:

Indeed, AlphaFold-generated predictions for structured domains in sA28 (56-146) (S7 Fig) are very similar to those determined by solution NMR spectroscopy, except that the predicted folded structure is not as compact as the NMR-defined structure in solution. In addition, AlphaFold predicted that A28 residues $D^{119}V^{120}I^{121}$ act as a flexible loop in a turn-like conformation (S7 Fig), rather than the short 3_{10}–helix resolved from the NMR structure (Fig 1F).

4. Methods: More information on how the virus fusion-from-without assay quantitation was conducted would be helpful. For instance, was the entire well imaged or random fields? How many fields?

In response to the reviewer's request, we have modified the text as shown below:

Line 629-644:

Vaccinia MV-triggered cell fusion-from-without under acidic pH

Cell-cell fusion assays triggered by VACV MV were performed as described previously [71] with slight modification. In brief, GFP- or RFP-expressing HeLa cells were mixed at a 1:1 ratio, seeded in 96-well plates, and cultured overnight. The next day, these cells were pre-treated with 40 µg/ml cordycepin for 1 hour at 37°C and subsequently infected with the inf/tnf cell lysates harvested from transient complementation assays. After 1 hour of incubation, the viral inoculum was removed and the cells were washed with warm PBS and then incubated in PBS (pH7.0) or PBS (pH5.0) at 37°C for 3 minutes. Complete growth medium was added to the cells to neutralize the acidic buffer and then replaced with fresh growth medium containing 40 µg/ml cordycepin. These cells were further incubated for 2 hours and subsequently photographed with a MD Image Xpress Micro XL system with a 20x objective. One field in each well was chosen randomly and imaged (596.7 x 596.7 µm) and fluorescence intensity was quantified. The percentage of cell fusion in each well was determined using Fiji software according to the equation: (Surface area of GFP^+RFP^+ double-fluorescent cells divided by the surface area of single-fluorescent cells) x100%. The experiments were independently repeated three times.

5. "(B)." at end of Figure 4 legend. Not needed?

Removed.

Reviewer #3:

The protein nomenclatures of VACV are not identical to mpox counterparts. E.g., A28 in VACV is A30 in mpox virus. Therefore, it may be necessary to modify the protein names of mpox virus involved in the paper or provide additional explanations in introduction section.

Indeed, A28 protein in vaccinia virus is A30 in mpox virus. H2 protein in vaccinia virus is still called H2 in mpox virus. The text has been modified accordingly in the Results (Line 170, 172, 173, 386), Materials and Methods (Line 461, 464 and 471) and the Legend of Fig 3 (Line 689-691 and 696).

2. The secondary structure of A28 is inconsistent in the text and the figures: the α3-helix in Figure 1C is forgotten to label, and the β5-strand is present in Figure 1C and 1D but is not described or missing in the text (lines 357 and lines 581-584) and Figure 4.

We thank the reviewer for pointing out these mistakes. The manuscript has been modified as described below:

The α3 helix is labeled in Fig 1C and 1D.

The β5-strand are included in the text as shown below:

Line 114-115:

and five anti-parallel β-strands-β1 (74-78), β2 (82-87), β3 (90-96), β4 (100-103) and β5(124-126)—with the remaining residues forming connecting loops (Fig 1C).

Line 128:

β-strands: 74−78, 82−87, 90−96, 100−103 and 124-126.

Line 660-661:

five β-strands: β1 (aa 74–78), β2 (aa 82–87), β3 (aa 90–96), β4 (aa 100–103) and β5 (aa 124–126);

3 I noticed that the SDS-PAGE-gel and immunoblot images in the manuscript were cropped to a single lane with small size, which makes almost all the images in figure panels spliced. Therefore, the authors may need to include the uncropped original images of these figures in supplementary materials.

All the uncropped original images of protein staining in SDS-PAGE gels (S3 Fig) and the images of immunoblots (S10-S15 Fig) are now included in the manuscript as described below:

S3 Fig. Coomassie staining of purified sA28 mutant proteins in SDS-PAGE gels.

Coomassie blue staining of all the recombinant sA28 mutant proteins after purification.

S10 Fig. Uncropped original images of Fig 5D.

S11 Fig. Uncropped original images of Fig 5E.

S12 Fig. Uncropped original images of Fig 6B.

S13 Fig. Uncropped original images of Fig 6C.

S14 Fig. Uncropped original images of Fig 7B.

S15 Fig. Uncropped original images of Fig 12B.

4. The scale bars should be added to these cell-cell fusion images: Figure 5B, Figure 6B, Figure 7B, and Figure 10B.

In the modified manuscript we have combined the cell fusion images of wild type and all the A28 mutants into one figure - Fig 5B with scale bars of 100 μm.

5. In Figure 8A and 8B, the vertical axes of some differential power curves lack scale unit values.

We thank the reviewer for pointing out the mistake which is corrected in Fig 8A and 8B of the revised manuscript.

6. The authors discussed that some anti-A28 antibodies may interfere with H2 binding to A28 through steric hindrance (lines 385-390). While previously studies have shown that the neutralizing antibody response could be enhanced when A28 and H2 are used together as a complex (Shinoda, K. et al., Virology, 2010, 405, 41-49). Therefore, more discussion should be included in the manuscript.

Following the reviewer's suggestion, we modified the discussion and included the above reference in the revised manuscript as shown below:

Line 436-441:

A previous study showed that the A28-H2 complex formation enhances the neutralizing antibody response raised by A28 protein alone [54], presumably by stabilizing an immunogenic conformation of A28. Although we only observed minor structural alteration of sA28 protein upon its binding to sH2 in CD analysis (Fig 9) and MD simulation (S5 Fig), these observations may imply a possibility that the A28-H2 interaction facilitates the exposure of neutralizing epitope(s).
